# Les urgences chirurgicales néonatales à l’hôpital provincial général de référence de Bukavu en République Démocratique du Congo

**DOI:** 10.11604/pamj.2016.24.219.8495

**Published:** 2016-07-12

**Authors:** Fabrice Cikomola Gulimwentuga, Alain Namugusha Kabakuli, Aline Bedha Ndechu, Georges Kuyigwa Toha, Yvette Lufungulo Bahati, Jeff Kabinda Maotela

**Affiliations:** 1 Département de Chirurgie, Hôpital Provincial Général de Référence de Bukavu et l’Université Catholique de Bukavu, République Démocratique du Congo; 2 Département de Pédiatrie, Hôpital Provincial Général de Référence de Bukavu et l’Université Catholique de Bukavu République Démocratique du Congo; 3 Ecole Régionale de Santé Publique de l’Université Catholique de Bukavu République Démocratique du Congo

**Keywords:** Urgences, chirurgicales néonatales, mortalité, Emergencies, neonatal surgery, mortality

## Abstract

**Introduction:**

L’objectif de cette étude était de déterminer la prévalence des urgences chirurgicales en néonatologie et en déterminer les facteurs épidémiologiques.

**Méthodes:**

Il s’agissait d’une étude de cohorte historique de 30 cas sur 3 ans allant de janvier 2010 en Décembre 2013, réalisé dans le département de chirurgie de l’Hôpital Provincial Général de Référence de Bukavu.

**Résultats:**

Les urgences chirurgicales néonatales représentent 1,31% pathologies chirurgicales en général. L’âge de consultation le plus fréquemment rencontré est de moins de 8 jours. La prédominance masculine a été notée avec un sex-ratio 3/2. Les pathologies les plus fréquemment rencontrées sont les urgences du tube digestif à 43,3 % suivies de celles neurologiques à 40%. 80 % des nouveau-nés ont bénéficié d’une intervention chirurgicale. Le taux de mortalité a été évalué à 43,3 %.

**Conclusion:**

Les pathologies chirurgicales néonatales existent dans notre milieu. Que les cliniciens soient attentifs et arrivent premièrement à poser le diagnostic avant la naissance et deuxièmement à bien prendre en charge dans le but de sauver la vie de ces enfants.

## Abstract (en)

**Introduction:**

This study aims to determine the prevalence of neonatal surgical emergencies and their epidemiology.

**Methods:**

This retrospective cohort study reports 30 cases whose data were collected in the Department of Surgery at the Bukavu Provincial General Reference Hospital over a period of 3 years, from January 2010 to December 2013.

**Results:**

Neonatal surgical emergencies account for 1.31% of surgical pathologies in general. The most frequent age of patients during medical consultation was less than 8 days. Male prevalence was noted with sex-ratio 3/2. The most frequent pathologies were gastrointestinal tract emergencies (43.3%) followed by neurological emergencies (40%). 80% of newborns underwent surgery. Mortality rate was 43.3%.

**Conclusion:**

Neonatal surgical pathologies occur in our midst. Clinicians should be on alert and implement prenatal diagnostic programs and proper neonatal care management in order to save the lives of these children.

## Introduction

Les urgences chirurgicales néonatales regroupent les affections du nouveau-né, qui se manifestent de la naissance à la fin du premier mois de la vie et qui nécessitent un traitement chirurgical urgent ; ce sont des anomalies des structures ou des fonctions, dont les troubles métaboliques, présentes a la naissance [1, 2]. Les urgences néonatales peuvent être liées à une anomalie congénitale ou encore être consécutive à une pathologie acquise d’expression éventuellement plus tardive. Elles regroupent principalement les urgences neurologies, les thoraciques, les abdominales, les uro-génitales et les pariétales.

Certaines urgences sont évidentes dès la naissance rendant ainsi le diagnostic aisé. Cependant, le diagnostic prénatal a totalement modifié les conduites à tenir face à une malformation congénitale, chirurgicalement curable ou non [2].

D’autres sont de révélation plus tardive rendant la démarche diagnostique nécessiteuse d’une mise en condition et des bilans plus approfondis. Si dans le pays industrialisés le pronostic vital s’est amélioré du fait du diagnostic anténatal, dans le pays en voie de développement la prise en charge se heurte à plusieurs difficultés [2–4].

Parmi les rares enquêtes disponibles, celle réalisée de 1992 à 2001 dans le service de chirurgie pédiatrique du Centre hospitalier Universitaire (CHU) de DONKA en République de Guinée, les urgences chirurgicales néonatales ont été évaluées à 4,22% des urgences chirurgicales en général. En 10 ans ils ont enregistré 37,39% des cas intéressant la paroi abdominale, 32,88% d’affections neurologiques, 27,48% concernant le tube digestif et enfin 2,25% pour les uro-génitale [2] l’étude menée de 1999 en 2006 dans le service de chirurgie pédiatrique du CHU ARISTIDE LE DANTEC de Dakar rapporte que la pathologie la plus fréquente à 34% était les malformations ano-rectales [3]. Le principal motif de consultation retenu au cours de cette étude au CHUA-JRA ANTANANARIVO MADAGASCAR de 2008 à 2009 relève une prédominance des urgences digestives chiffrées à 39% suivies par ordre de fréquence ; des urgence pariétale (17%), des urgences thoraciques (5%) et des urgences uro-génitales (3%) [5].

La place et les problématiques des urgences chirurgicales néonatales dans les pays en voie de développement ne sont pas bien connues. Aussi, nous sommes-nous permis d’entamer ce travail, une première dans notre milieu hospitalier. Il s’est étendu sur trois ans: de 2010 à 2013.

Le principal objectif poursuivi par ce travail est d’étudier la prévalence des urgences chirurgicales néonatales dans le département de chirurgie de l’hôpital Provincial Général de Référence de Bukavu.

## Méthodes

### Type et cadre d’étude

Notre étude était une cohorte historique, elle s’inscrit dans la période allant du 1^er^ janvier 2010 au 31 décembre 2013.

Le cadre de notre étude était l’Hôpital Provincial Général de Référence de Bukavu (HPGRB) qui se situe dans la ville de Bukavu, chef-lieu de la province du Sud-Kivu, elle-même située à l’Est de la République Démocratique du Congo, entre 2°31’ de latitude sud et à 28°50’ de longitude Est et est séparée du Rwanda par le lac Kivu (1460 m d’altitude) et la rivière Ruzizi. Administrativement, elle est subdivisée en trois communes : Ibanda, Kadutu et Bagira. Sa population a été estimée à 619 916 hab. en 2008. Les 3 communes sont composées de 20 quartiers dans lesquels un total de 322 avenues est répertorié. Cinquante-huit pourcent de la population générale sont constitués des sujets de moins de 20 ans alors que 42% sont âgés de 20 ans et plus. La ville connait un climat des montagnes avec 25°C en saison chaude et à 15°C en saison de pluie. Hôpital Provincial Général de Référence de Bukavu est construit selon le type pavillonnaire et possède 17 pavillons avec une capacité d’accueil de 358 lits. Il comprend six principaux départements dont celui de chirurgie dans lequel nous avons effectué la présente étude et les autres départements sont : la médecine interne, la pédiatrie, la gynécologie-obstétrique, la biologie et anatomo-pathologie et enfin les spécialités.

Le département de chirurgie actuellement est organisé en quatre services : traumatologie et orthopédie, chirurgie digestive, urologie et neurochirurgie.

### Population

Notre population d’étude était composée de nouveau-nés de 1 à 30 jours de vie ayant été hospitalisés à l’HPGRB pour une pathologie chirurgicale malformative opéré ou non, et répondant à nos critères d’inclusion. Un dossier d’hospitalisation a été élaboré pour chacun de nos patients.

### Critères d’inclusion

Les critères de sélection des cas ont retenu : tout nouveau-né : agé de 1 à 30 jours; hospitalisé pour une pathologie chirurgicale néonatale ayant nécessité une prise en charge immédiate; le dossier du malade doit fournir le maximum de renseignements nécessaires à l’étude. Pour chaque dossier ont été relevées les données suivantes : l’âge, le sexe, la maturité gestationnelle, le milieu d’origine, le mode de consultation, la période du diagnostic les imageries réalisées, le diagnostic retenu, la prise en charge, l’évolution et complications présentées en post opératoire.

### Critères de non-inclusion

Les nourrissons et enfants de plus de 30 jours de vie et les dossiers comportant peu de données en vue de minimiser les facteurs d’erreurs.

### Analyse des données

Les paramètres suivants ont été analysés : âge en jours, sexe, maturité gestationnelle, origine géographique, le mode d’admission, la période du diagnostic, les imageries réalisées, les pathologies rencontrées, la prise en charge, les complications présentées au cours de l’hospitalisation et en fin l’issu.

Pour l’analyse de données, nous avons eu recours au logiciel d’Epi info.3.5.4. Nous avons fait les statistiques descriptives usuelles. La comparaison des proportions a bénéficié de test de khi-carré de Pearson ou le test de Fisher selon le cas. Le seuil de signification retenu était à 0,05.

## Résultats

Notre étude a pu recenser 30 cas sur 2287 cas admis aux urgences et représentant que 1,31% des admissions dans le département de chirurgie. Aucun nouveau-né de notre étude n’a cependant bénéficié d’un diagnostic anténatal.

Les enfants de sexe masculin représentaient une fréquence de 60 % de notre échantillon, soit un sex-ratio de 3 garçons sur 2 filles. La majorité de ces enfants proviennent de communes défavorisées et de moins nanties de tendance culturelle urbano-rurale, Kadutu et de celle de Bagira à des fréquences respectives de 46,7% et 26,7%. Les nouveau-nés matures prédominaient avec fréquence 73,3% de notre échantillon par rapport aux prématurés (Tableau 1).

**Tableau 1:** Le profil des nouveau-nés avec urgences néonatales chirurgicales selon l’âge de consultation

	n	%
**Sexe**		
F	12	40,0
M	18	60,0
**Age (jour)**		
0-8 jours	18	60,0
9-30 jours	12	40,0
**Provenance**		
Bagira	8	26,7
Ibanda	4	13,3
Kadutu	14	46,7
Périphérie	4	13,0
**Maturité gestationnelle à la naissance**		
A terme	22	73,3
Prématurité	8	26,7
**Mode d’admission**		
HPGRB+	17	56,7
Centres périphériques	13	43,3

+ HPGRB=Hôpital Provinciale Général de Référence de Bukavu

L’âge médian d’admission était à 10 jours avec un minimum de quelques heures et un maximum de 3 jours.

**Figure 1:**
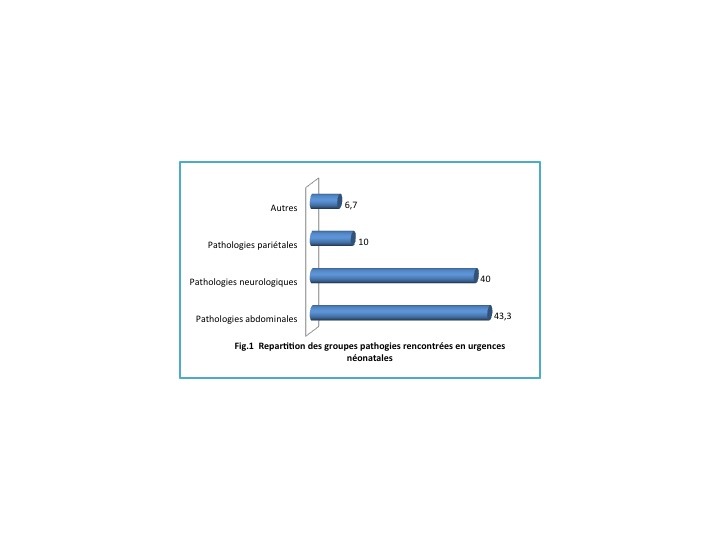
Répartition des groupes pathologies rencontrées en urgences néo natales

Dans notre étude les pathologies abdominales sont les plus fréquentes, elles représentaient 13 nouveau-nés sur les 30 retenus soit 43,3% suivies des pathologies neurologique à une fréquence de 12 cas soit 40% (Figure 1).

**Figure 2:**
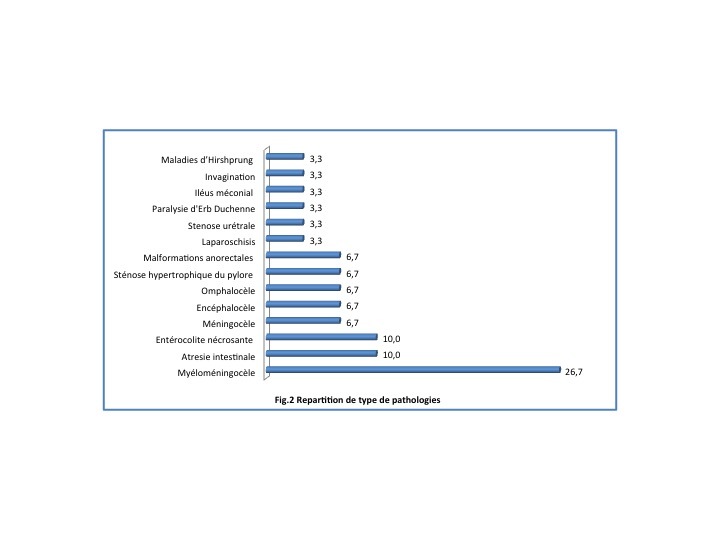
Répartition des types des pathologies

Parmi les pathologies abdominales, les occlusions intestinales aigues sur atrésie intestinale (3 malades), entérocolite nécrosante (3 malades), sténose hypertrophiques du pylore (2 malades) étaient plus fréquentes mais dans l’ensemble de toutes les pathologies d’urgences néonatales, ces trois pathologies représentent 10% chacun pour l’atrésie intestinale et entérocolite nécrosante comme le montre la Figure 2. Pour les pathologies neurologiques, la plus fréquente rencontrée était spina bifida type myéloméningocèle (8 patients), spina bifida type méningocèle (2 malades) qui représentent respectivement 26,7% et 6,7% dans l’ensemble tandis que elles ont une fréquence de 66,7% et 16,7% dans les pathologies neurologiques. Les pathologies pariétales étaient représentées par 2 malades avec omphalocèle et 1 malade avec laparoschisis. Le groupe « autres pathologies », nous avons mis un malade avec sténose urétrale sur tumeur vésicale avec le rein droit poly kystique (1 malade), paralysie d’Erb Duchenne (1 malade).

Dans notre série pour appuyer le diagnostic clinique, on a eu recours selon les cas soit à l’échographie (13 malades) soit à radiographie (12 malades). Les complications les plus fréquentes sont l’hydrocéphalie avec hypertension intracrânienne à une fréquence de 9 patients soit 30% (Tableau 2). Vingt-quatre nouveau-nés (80%) ont bénéficié d’une intervention chirurgicale. Treize de nos patients étaient décédés (43,3%) tandis que 7 étaient guéris (23,3%).

**Tableau 2:** Exploration et prise en charge

	n	%
**Imageries médicales**		
Echographie	13	43,3
Radiographie	12	40,0
Aucune	5	16,8
**Prise en charge**		
Mesure de réanimation	6	20,0
Traitement chirurgical	24	80,0
**Complications**		
Péritonite	5	16,7
MPC++	2	6,7
Hydrocéphalie et HTIC^+++^	9	30,0
Aucune	6	20,0
Détresse respiratoire	3	10,0
Sepsis	2	6,7
Défaillance multi viscérale	2	6,7
Sepsis et hydrocéphalie	1	3,3
**EVOLUTION**		
Décès	13	43,3
Perdu de vue	10	30,0
Guéri	7	23,3

++ MPC=Malnutrition Protéino-calorique ; HTIC=Hypertension Intracrânienne

La mortalité précoce était prédominante à une fréquence de 11 cas sur 18 nouveau-nés de moins de 8jours soit 61,1%. Le taux de mortalité est élevé pour les urgences abdominales à 53,8%. L’issue des cas ayant bénéficié uniquement des mesures de réanimation était de 6 décès sur les 6 enregistrés soit 100%. Quant aux nouveau-nés qui ont pu bénéficier d’un traitement chirurgical, ils représentent une fréquence de 6 décès sur 24 (29,2%).

Les enfants de moins de 8 jours avaient un risque élevé (OR= 7,2 (IC à 95% 1,3-61,6) de mourir que les enfants de plus de 8 jours. Les enfants prématurés avaient un risque accru de mourir (OR=2,8) que les enfants nés mais sans signification statistique. La mortalité était statistiquement significative en association avec les complications et la prise en charge. En effet les enfants qui ont été prise en charge par une réanimation seulement étaient plus décédés par rapport aux autres pris en charge par la chirurgie. Il y a eu beaucoup de décès chez les enfants qui ont présenté les complications suivantes : Sepsis, Sepsis et HTIC, péritonite, défaillance multi viscérale, détresse respiratoire comme le montre le Tableau 3.

**Tableau 3:** Les déterminants de la mortalité due aux urgences néonatales

	Décès n(%)	Guéris/perdue de vue n(%)	p
**Age**			
Moins de 8 jours	11(61,1)	7(38,9)	0,03+
Plus de 8 jours	2(16,7)	10(83,3)	
**Age gestationnel**			0,38+
Prématurés	5(62,5)	3(37,5)	
Matures	8(36,4)	14(63,6)	
**Groupe des pathologies**			0,83
Abdominales	7(53,8)	6(46,2)	
Neurologiques	2(16,7)	10(63,6)	
Pariétales	3(100,0)	0(0,0)	
Autres	1(50,0)	1(50,0)	
**Complications**			0,03
Aucune	0(0,0)	6(100,0)	
Défaillance multi viscérale	2(100,0)	0(0,0)	
Détresse respiratoire	3(100,0)	0(0,0)	
Hydrocéphalie et HTIC	1(12,5)	8(87,5)	
Malnutrition proteino- énergétique	0(0,0)	2(100,0)	
Péritonite	4(80,0)	1(20,0)	
Sepsis	2(100,0)	0(0,0)	
Sepsis et HTIC	1(100,0)	0(0,0)	
**Prise en charge**			0,003+
Mesure de réanimation	6(100,0)	0(0,0)	
Traitement chirurgical	6(33,3)	18(66,7)	

+ Test de Fisher exact

## Discussion

Nous avons mené une étude sur la prévalence des urgences chirurgicales néonatales dans le département de chirurgie. Notre étude a des limites méthodologiques, la prévalence trouvée ne semble pas refléter la réalité de ce problème dans la région car il existe d’autres centres hospitaliers qui n’ont pas été considéré dans notre étude. Néanmoins cette étude donne une vue d’ensemble de cette pathologie en absence de données y afférentes.

Notre série a trouvé une prévalence de 1,31%, elle est inférieure à celle estimée par l’OMS en 2010 (6%) et cette dernière ne tenant pas compte de toutes les malformations congénitales [1] et inferieure à celle trouvée à Abidjan en 1997 (4,9%) par F.Coulibaly-Zerbo et al. [6]. La différence entre la prévalence trouvée dans notre étude et les autres peut-être expliquée par certaines raisons : certains cas n’ont pas été retenus pas défaut d’informations nécessaires pour notre étude, d’autres malades vus dans les centres hospitaliers périphériques ne sont pas transférés à l’HPGRB alors qu’elle est la seule structure où nous avons eu à sélectionner notre échantillon. D’autres malades ont pu être méconnus à la naissance d’où n’ont pas eu à bénéficier d’une consultation chirurgicale. Tous ces éléments pris en compte expliqueraient la différence entre nos chiffres et ceux qu’ont trouvés certains auteurs africains [7].

Concernant l’âge à l’admission, l’étude a trouvé une moyenne de 10 jours alors que NDOUR O. et al. au Mali en 2009 [4] avait trouvé 8 jours et M.F RALAHY et al. à ANTANANARIVO en 2010 [5], 2,5jours. Soixante et un pourcent d’enfants étaient dans la tranche d’âge entre 1 à 8 jours alors que NDOUR avait 68% dans la même tranche d’âge tandis que Keita M. et al. Mali en 2006 [3] avait dans la tranche entre 7 à 28 jours 68.8%. La précocité de consultation dans notre étude se justifierait par le fait que la majeure partie des nouveau-nés de notre échantillon (60%) provient directement du service de néonatologie de la structure hospitalière où a eu lieu l’étude. La présence d’une équipe médicale avisée dans l’unité de néonatologie ainsi que la disponibilité d’un service d’imagerie permet le diagnostic précoce. Cependant nous sommes loin de l’âge moyen de 1 jour devenu courant et impératif dans les pays industrialisés suite au diagnostic anténatal [1, 4–6, 8–10].

Selon la littérature ces pathologies sont beaucoup plus fréquentes chez le garçon que chez la fille [1, 6, 10–14]. Notre étude est dans cette logique avec un sex ratio à 1.5 comme celui de Keita [3] contrairement à ce que M.F RALAHY et al. [5] a trouvé 2.12. L’explication scientifique à la prédominance masculine de ces pathologies du nouveau-né reste obscure quant à ce qui est de notre étude.

Pour la série de Dakar par NDOUR O. et al, le plus grand nombre des patients provenait de la banlieue de Dakar (65% de cas) suite à l’absence d’unités de chirurgie pédiatrique dans plusieurs hôpitaux et ceux provenant des régions de l’intérieur du pays ne représentaient que 25% de cas. Ils l’expliquent par un défaut des moyens d’évacuation appropriés. Dans notre série la majeure partie de nos patients provenait de la banlieue de Bukavu (quartiers de Kadutu et de Bagira : 73,4% des cas) et 4 patients (13,3%) provenaient de différents territoires de l’intérieur de la province. Le manque de transport adéquat pour l’évacuation des nouveau-nés pathologiques des territoires de l’intérieur de la province ainsi que la méconnaissance de ces pathologies par certains praticiens dans les centres hospitaliers de l’intérieur de la province seraient à la base d’un faible taux de transfert de la périphérie.

Pour I. Ouédraogo et al, la prématurité, l’existence des malformations associées et le faible poids de naissance sont de facteur de risque de mortalité. La série de COULIBALY-ZERBO F. et al, à Abidjan, 1997, 5 nouveau-nés sur 27 soit 19% étaient des prématurés. Dans la littérature 1/3 des nouveau-nés présentant une malformation sont des prématurés [13–15]. Pour notre étude 22 nouveau-nés sur 30 sont issus d’une grossesse à terme tandis que 26,7% sont des prématurés soit environ 1/3 de notre population d’étude. Dans le pays développé, la prématurité n’est plus un facteur de mauvais pronostic [4, 6, 13, 14]. Par contre dans notre pays la prématurité est encore l’une des causes de la mortalité néonatale élevée et constitue de ce fait un facteur de mauvais pronostic [3].

Les urgences chirurgicales du nouveau-né comprennent essentiellement les urgences néonatales liées à une anomalie congénitale. Ces malformations congénitales devraient bénéficier d’un diagnostic prénatal par un examen échographique ou milieu par l’imagerie à résonance magnétique (IRM) pour que l’accouchement se déroule dans un centre spécialisé de réanimation et de chirurgie néonatale [1, 8, 10, 14–19]. Dans notre milieu nous n’avons pas eu le diagnostic prénatal de nos patients.

A ce qui concerne les pathologies rencontrées : les pathologies abdominales sont les plus grandes pourvoyeuses des urgences chirurgicales néonatales dans notre série suivie de pathologies neurologiques comme dans d’autres études. Le taux de mortalité liée aux urgences chirurgicales abdominales néonatales avoisine celui de l’étude d’AGUENON A.R. et al qui était évalué à 58% [13]. M.F RALAHY par contre note un taux de mortalité de 16% en ce qui concerne les pathologies abdominales pour sa série [5]. Les pathologies neurologiques qui se répartissent par ordre de fréquence: myéloméningocèle, méningocèle et encéphalocèle. Cette répartition rencontre les données de la littérature qui décrit une prédominance des myéloméningocèle [2, 10, 14, 16, 18, 19]. Pour les pathologies pariétales nous avons eu un laparoschisis et deux omphalocèles. Comme dit plus haut aucun de nos patients n’a bénéficié d’un diagnostic échographique en anténatal comme dans la série de M.F RALAHY [5] ainsi que celle de NDOUR [4]. Pourtant la littérature actuelle parle d’un diagnostic prénatal par une échographie endovaginale précoce déjà entre la 12ème et la 14ème semaine d’aménorrhée pour les anomalies de la paroi abdominale. Au troisième trimestre l’échographie peut même préciser la vitalité des anses intestinales pour le laparoschisis ainsi que la taille et le contenu d’une omphalocèle [1, 5, 12, 16, 18].

Concernant l’issue 43.3% de nos patients étaient décédés, ce taux est inférieur à celui trouvé par NDOUR [4] 68% mais était supérieur à celui de 41% de KOURA à Cotonou en 1993 [18] et 33.3% DOUMBOUYA N. au Mali [11, 19]. Tous ces auteurs justifient le taux de mortalité élevé par : la fragilité des nouveau-nés, le diagnostic qui n’est pas posé en période prénatale, par l’absence d’une unité de réanimation néo-natale spécifique, le retard de consultation, le fait que le nouveau-né est vu pendant qu’il présente déjà certaines complications, l’approvisionnement en médicaments qui est laissé à la seule charge des parents et un mode d’évacuation des nouveau-nés défectueux. Ces faits rencontrent exactement la réalité de chez nous, ce qui explique le taux de mortalité élevé de notre série.

## Conclusion

Au bout de notre étude qui a porté sur les urgences chirurgicales néonatales à l’HPGRB durant une période de 3 ans nous retenons que les urgences néonatales sont fréquentes avec une moyenne de 10 cas par an. L’âge de consultation et le sexe le plus touché sont les mêmes que ceux de la littérature. Aucun de nos patients n’a bénéficié d’un diagnostic prénatal et les pathologies les plus fréquentes sont abdominales suivies de celles neurologiques. Le taux de mortalité est très élevé à 45,3% de nouveau-nés pathologiques et les facteurs influençant ce taux élevé sont : le jeune âge, les complications survenues, l’absence de prise en charge chirurgicale. Il est important que les praticiens s’exercent à rechercher ces genres de pathologies et que les unités appropriées de diagnostic, de prise en charge et de suivi soient mise en place.

### Etat des connaissances actuelles sur le sujet

- Dans les pays industrialisés, le diagnostic prénatal par les examens d’imageries a actuellement modifié les conduites à tenir face à une malformation congénitale;
- Avec une prévalence de 6% selon l’OMS, leur taux de mortalité reste très élevé en Afrique pouvant aller jusqu'à 70 %.

### Contribution de notre étude à la connaissance

- Notre étude donne, a la face du monde, une vue d’ensemble sur la prévalence des urgences chirurgicales néo natales dans notre région;
- Comme pour les autres auteurs africains, les pathologies abdominales sont les plus grandes pourvoyeuses des urgences chirurgicales néonatales avec un taux de mortalité avoisinant 50%, l’échographie endovaginale peut être introduite dans la démarche diagnostique afin de diminuer ce taux de mortalité;
- Nos résultats interpellent les cliniciens de la région; la prise en charge pouvant commencer en anténatale par l’échographie endo vaginale, le geste chirurgical posé en urgence peut réduire la mortalité.
